# Left Pulmonary Artery Coarctation Associated with Pneumonia and Pulmonary Hypertension in a Cat

**DOI:** 10.3390/vetsci8120325

**Published:** 2021-12-12

**Authors:** Carlotta Valente, Massimiliano Tursi, Helen Poser, Carlo Guglielmini

**Affiliations:** 1 Department of Animal Medicine, Production and Health, University of Padova, Viale dell’Università 16, 35020 Padova, Italy; carlotta.valente@unipd.it (C.V.); helen.poser@unipd.it (H.P.); 2 Department of Veterinary Sciences, University of Turin, Largo Paolo Braccini 2, 10095 Grugliasco, Italy; massimiliano.tursi@unito.it

**Keywords:** congenital heart disease, echocardiography, feline, right ventricular hypertrophy

## Abstract

A five-month-old European shorthair female kitten was referred because of recurrent episodes of respiratory distress. Results of physical examination, thoracic radiography, and echocardiography led to a presumptive diagnosis of severe precapillary pulmonary hypertension (PH) and interstitial pneumonia associated with right-sided cardiac remodeling. The cat rapidly died because of respiratory insufficiency. Pulmonary and cardiovascular pathological findings evidenced left pulmonary artery coarctation, severe right-sided cardiac hypertrophy, and bilateral pneumonia. This is the first report of pulmonary artery coarctation associated with pneumonia and PH in a cat.

## 1. Introduction

Obstructions of the pulmonary arteries (PAs) are usually congenital disorders involving the pulmonary trunk, the main right or left PA, or the peripheral (i.e., segmental and subsegmental branches) PAs which are rarely observed in cats [1]. Stenosis of the pulmonary trunk or branch PAs are less common than pulmonary valve stenosis or infundibular stenosis and include PA interruption, also known as unilateral absence or atresia of the PA, PA stenosis, peripheral or branch PA stenosis, and coarctation of the PA [2]. In humans, the stenosis of the PA branches is classified as four forms: type I occurs as a single constriction of varying length involving the main, left or right PA; type II stenosis occurs at the PA bifurcation and involves the distal end of the main PA and the origins of the left and right PAs; type III stenosis involves multiple segmental PAs at their ostium with concurrent post-stenotic dilatation; and type IV stenosis involves either peripheral segments and central PAs [1]. In cats, only type I and type II forms have been described [3,4].

Pulmonary hypertension (PH) is a pathophysiological disorder characterized by increased pressure in the pulmonary arterial vascular bed that can be associated with different cardiovascular and respiratory disorders. In humans, five different types of underlying pathophysiological mechanisms have been recognized for the clinical classification of PH, namely pulmonary arterial hypertension (type 1 PH), PH due to left heart disease (type 2 PH), PH due to lung diseases and/or hypoxia (type 3 PH), chronic thromboembolic PH and other PA obstruction (type 4 PH), and PH due to unclear and/or multifactorial mechanisms (type 5 PH) [5]. In dogs, a recent classification of PH has added an additional type of PH, specific for dogs, namely PH due to parasitic disease (e.g., heartworm disease) considered as type 5 PH [6]. Thus, PH due to unclear and/or multifactorial mechanisms has been reclassified as type 6 [6]. More generally, the hemodynamic clinical classification distinguishes pre-capillary PH, characterized by increased pulmonary artery pressure (PAP) and normal pulmonary artery wedge pressure ([PAWP], i.e., type 1, 3, 4, 5, and 6 PH) from post-capillary PH, in which both PAP and PAWP are increased (i.e., type 2 and 6 PH) [6]. In cats, PH is uncommonly reported and, with the exception of a recent case series of 22 cats with post-capillary PH secondary to left-sided congestive heart failure [7], the majority of reports are single case descriptions of PH associated with congenital cardiovascular disease [8,9,10], pulmonary fibrosis and chronic upper airway obstruction [11,12], pulmonary thromboembolism [13], parasitic diseases [14,15], and pulmonary capillary hemangiomatosis [16]. 

To the best of our knowledge, only one case of juxtaductal coarctation of both main PAs not associated with PH has been reported in the feline literature [4]. We describe here a case of a cat presenting left PA coarctation associated with pneumonia, severe right-sided cardiac remodeling, and pre-capillary PH. 

## 2. Case Description

A five-month-old, female, domestic shorthaired cat, weighing 1.65 kg, was referred to the Veterinary Teaching Hospital of the University of Padua with a two-month history of recurrent episodes of respiratory distress. The cat had been treated in the previous 10 days with doxycycline 10 mg/kg PO q24 h and inhaled beclomethasone with only partial relief of the respiratory signs. 

On physical examination, the cat was alert but panting with open mouth breathing. The mucous membranes were pink-pale and the respiratory rate was 80 breaths/min with a restrictive respiratory pattern. Thoracic auscultation revealed respiratory crackles on both side of the thorax without appreciable cardiac murmur. The heart rate was 190 bpm. The laboratory work-up included serology for feline leukemia virus antigen and feline immunodeficiency virus (FIV) antibodies and both tests were negative, results of complete blood count were unremarkable. To minimize the respiratory distress, successive diagnostic procedures were performed with the cat under sedation with butorphanol (0.2 mg/kg IV), dexmedetomidine (5 μg/kg IV), propofol IV to effect, and oxygen delivery. 

**Figure 1:**
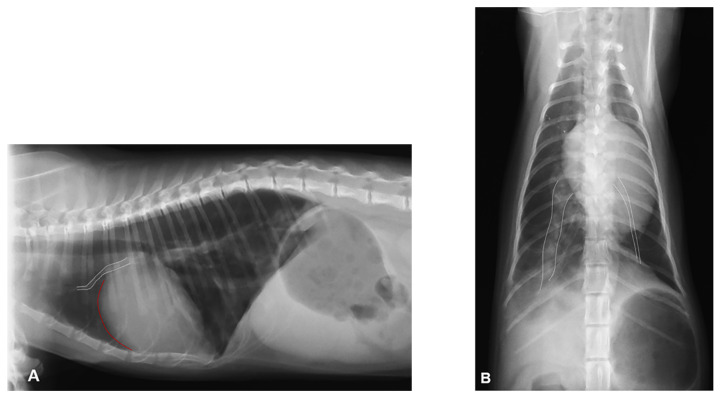
Thoracic radiography at presentation shoving an interstitial pulmonary pattern in the right caudal lung lobe: (**A**) Right lateral view showing cardiomegaly with increased convexity of the cranial cardiac border (red line) and prominent and tortuous right cranial pulmonary artery (white dotted lines). (**B**) Dorso-ventral view showing severe asymmetry of the caudal pulmonary arteries with severely enlarged and hypovascular right and left caudal pulmonary artery, respectively (white dotted lines).

Thoracic radiography showed subjective generalized cardiomegaly with prominent right-sided cardiac chambers and a vertebral heart score of 10.0 (Figure 1A,B). The right cranial and caudal pulmonary artery were prominent while the left pulmonary arteries were barely appreciable suggesting left lung hypoerfusion. In the lungs, an interstitial pulmonary pattern was found in the right caudal lung lobe. A gas filled stomach was an additional radiographic finding, likely due to aerophagia associated with respiratory distress. 

**Figure 2:**
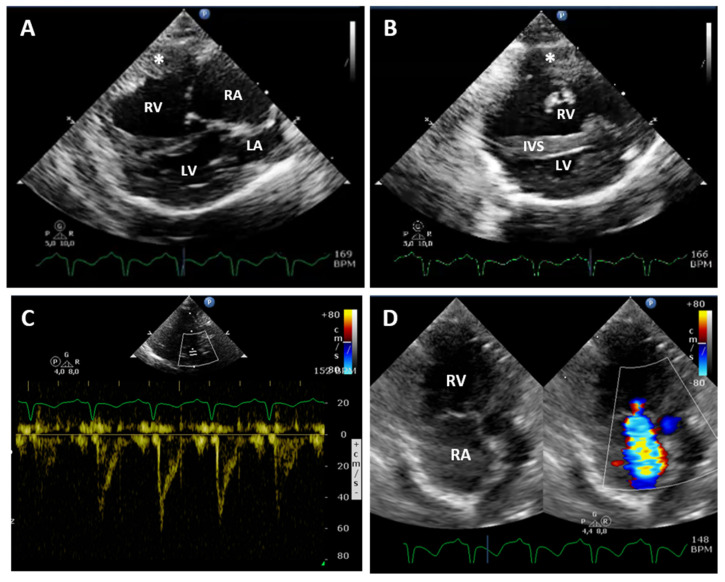
Echocardiography at presentation: (**A**,**B**) Right parasternal window long-axis 4-chamber view and short-axis view at the level of papillary muscles, respectively. Severe right atrium and ventricle dilation, right ventricle free wall hypertrophy and a reduced left ventricular diameter were evident. (**C**) Pulsed-wave Doppler tracing of the pulmonary artery systolic flow: the laminar flow profile is asymmetrical with a sharp peak, corresponding to type II pulmonary blood flow. (**D**) Left parasternal window apical view optimized for the right heart. On color flow mapping (on the right), moderate-severe tricuspid regurgitation is evident. RA, right atrium; RV, right ventricle; IVS, interventricular septum; LA, left atrium; LV, left ventricle; *, right ventricular free wall.

Trans-thoracic echocardiography revealed severe right atrial and ventricular dilatation and reduced left ventricular cavity (right atrial diameter = 1.5 cm; right ventricular diastolic diameter = 1.13 cm, left ventricular diastolic diameter = 0.56 cm) (Figure 2A,B). The right ventricular free wall was hypertrophic (diastolic thickness = 0.49 cm) and interventricular septal flattening was evident (diastolic interventricular septum thickness = 0.45 cm). The cardiac septa were intact, and the left atrium (LA) was not enlarged (LA to aortic root ratio = 1.36, normal value < 1.6). No obstruction of the right ventricular outflow tract was found. The pulmonic valve was normal, and the pulmonary blood flow was laminar with a sharp peak (type II pulmonary blood flow) on pulsed-wave Doppler interrogation, suggesting increased pulmonary artery pressure (Figure 2C). The pulmonary acceleration time (AT) and ejection time (ET) were 45 ms and 153 ms, respectively with an AT to ET ratio (AT/ET) of 0.29. Moderate-severe tricuspid regurgitation (TR) was found on color flow Doppler (Figure 2D) with a peak systolic velocity of 5.57 m/s corresponding to an estimated pressure gradient of 133 mmHg using a modified Bernoulli equation. The pulmonary trunk appeared dilated, but evaluation of the main PAs was not feasible. A bubble test was performed using 2 mL of saline solution with 0.5 mL of air agitated forcefully between two syringes connected with a three-way stopcock and rapidly injected in the left cephalic vein trough a 22G catheter. As no echo contrast was detected in the left heart and in the abdominal aorta, intracardiac or vascular extra cardiac right-to-left shunts were excluded. 

A presumptive diagnosis of severe right sided cardiac remodeling due to precapillary PH, left lung hypoperfusion, and pneumonia was made. In addition to pneumonia, other differential diagnoses for precapillary PH included distal PA stenosis, pulmonary parasitic disease, and pulmonary capillary hemangiomatosis. Selective angiogram and computed tomography scan were proposed to refine the diagnosis but were denied by the owner. The cat was discharged on doxycycline 10 mg/kg PO q24 h and sildenafil 0.5 mg/kg PO q12 h. Two days after our visit the cat was presented to the Emergency Service because of worsening of respiratory signs that prompted sudden death because of cardiorespiratory arrest.

**Figure 3:**
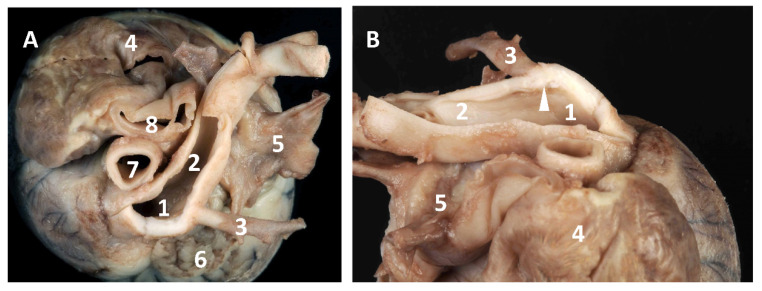
Gross pathology: (**A**) Dorsal view of the heart base showing severe dilatation of the pulmonary trunk (1) and right pulmonary branch (2), severe hypotrophy of the left pulmonary branch (3), and severe dilatation of the right atrium and auricle (4); 5: left atrium with pulmonary veins; 6: left auricle; 7: ascending aorta; 8: cranial vena cava; (**B**) Right lateral view of the heart base after opening of the pulmonary artery showing atresia of the origin of the left branch (arrow). The same numbers identify the same structures as in Figure 2A.

On gross pathology, multifocal, compact, and brownish-yellow pulmonary areas associated with severe reactive enlargement of the tracheobronchial lymph nodes were found. The heart demonstrated severely increased right ventricular volume and severe dilatation of the right atrium and auricle. The PA was markedly dilated at the level of the main trunk and right branch, with severe and diffuse hypoplasia of the left branch (Figure 3A). The examination of the PA cavity showed an atresia of the origin of its left branch (Figure 3B). The right ventricular outflow tract and tricuspid valve had no lesion. The cross section of the ventricles showed severe concentric right ventricular hypertrophy with a thickness of the free wall and interventricular septum of about 0.6–0.7 cm and 0.5–0.6 cm, respectively.

**Figure 4:**
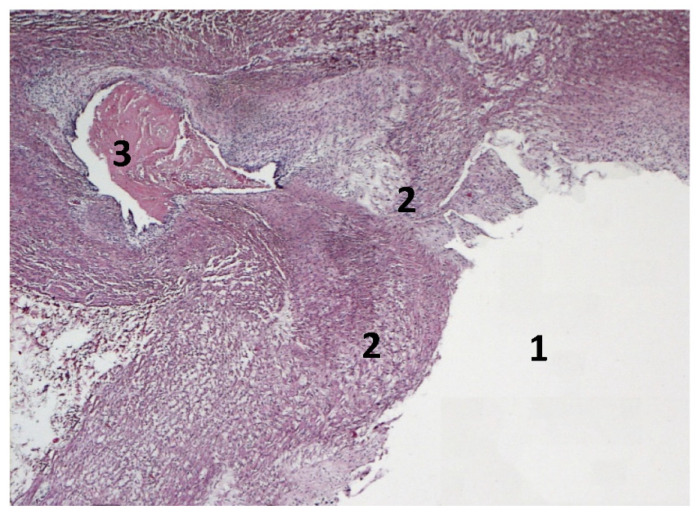
Histopathological image of the longitudinal section of the origin of the left pulmonary artery branch with its lumen (1). A thick layer of fibromyxoid tissue along the endothelium completely occludes the origin of the artery (2). In the residual cavity of the left branch, there is a voluminous thrombus (3). Hematoxylin & eosin stain. 2.5X.

Multiple samples of lung, tracheobronchial lymph nodes, right ventricle, and left PA branch were examined histologically according to routine techniques. In the right lung, severe macrophagic pneumonia, characterized by alveolar infiltration of activated macrophages with wide and granular cytoplasm often containing optically empty vacuoles, and eosinophilic granular material was found. In the left lung, multifocal and severe lymphocytic interstitial infiltrates and a mild mid-intimal hypertrophy of medium-caliber arteries were present. Severe reactive hyperplasia of the tracheobronchial lymph nodes was an additional finding. The right ventricle showed severe hypertrophy of myocardiocytes with multifocal and minimal interstitial fibrosis. The longitudinal section of the PA showed complete atresia and occlusion of the origin of the left branch by fibromyxoid tissue along the endothelial layer. The left branch of the PA had a voluminous thrombus in the residual cavity of its first portion (Figure 4).

The final diagnosis was left PA coarctation associated with pneumonia, pre-capillary PH, and severe right-sided cardiac remodeling. 

## 3. Discussion

Arterial coarctation represents a segmental retraction of the arterial vascular wall with a consequent reduction of its lumen [17]. In particular, juxtaductal PA coarctation indicates the congenital obstruction near the insertion of the arterial ligament, which is the remnant of the closure of the ductus arteriosus [17,18]. During the physiological closure after birth of the ductus arteriosus, the smooth muscles of the duct contract also involving the portion incorporated in the aortic or pulmonary wall. The progressive formation of the arterial ligament will follow [19]. The development of PA coarctation have two possible explanations. First, the dilation of the pulmonary outflow tract can cause a loop of the PA branch, creating a fibrous ridge with a subsequent obstruction at the point of ductal insertion. Second, the migration of ectopic ductal tissue along the pulmonary artery wall can cause a consequent juxtaductal coarctation [18,19,20]. Both the right and left ductus arteriosus are present in fetal life. Progressively, the right-sided should regress, even if, rarely, both sides remain patent [21]. Ductal atresia can induce PA occlusion and impaired pulmonary vasculature growth associated with pulmonary artery hypoplasia [22]. In the cat reported here, the presence of fibromyxoid tissue at the origin of left PA branch supports the hypothesis of a progressive migration of left-sided ductal ectopic tissue in the wall of the left PA with consequent coarctation and atresia. These findings are typical of type I PA stenosis [3]. In the previously described case of feline PA coarctation [4], the smaller size of the homolateral lung lobes supported the hypothesis that the complete occlusion of the left PA occurred early in life leading to functional severely reduced left-sided pulmonary blood flow. In the cat here described, no alteration of lung’s size, particularly the left one, was found. Therefore, we speculate that the complete closure of the origin of the left branch PA occurred progressively and not immediately after birth, without a marked neonatal impairment of the homolateral pulmonary vascular bed. At necropsy, careful research of collateral blood vessels bypassing the obstruction at the origin of the left branch PA was unsuccessful, thus excluding an alternative blood supply to the left lung. Therefore, it is likely that the blood flow reduction at the origin of the left branch PA occurred progressively in the time frame from birth to death. A progressive mechanism of pulmonary ductal coarctation and left PA interruption has also been recently recognized in humans with localized narrowing or even interruption of the pulmonary arteries [19]. In particular, the neural crest-derived muscular ductus arteriosus may continue contraction and stenosis formation even after birth. This process can be responsible of postnatal development of proximal left PA interruption [19]. Selective angiography and thoracic CT are the most useful diagnostic tools for precisely depicting the position and severity of any supravalvular pulmonic stenosis [6]. In the cat described here, these procedures were not conducted because of owner’s denial. Careful examination of thoracic radiographs evidenced an asymmetric distribution of PA blood flow with left lung hypoperfusion but the final diagnosis of left PA coarctation was only obtained at post-mortem examination.

The development of PH and associated severe right-sided cardiac remodeling was an additional feature of the cat reported here. Although direct measurement of PA pressure was not performed, the clinical (i.e., respiratory distress at rest) and echocardiographic findings strongly supported the diagnosis of PH, according to the recent ACVIM guidelines established for dogs [6]. In particular, the presence of a Doppler-estimated PA pressure of at least 133 mmHg was associated with involvement of three anatomic sites, namely the ventricles (i.e., right ventricular hypertrophy, interventricular septal flattening, and decreased size of the left ventricle), the PA (i.e., dilated PA, asymmetric spectral Doppler pulmonic flow profile with a sharp peak, AT < 52 ms and AT/ET < 30), and the right atrium that was severely dilated. Pre-capillary PH was then diagnosed because of the absence of echocardiographic features of left-sided cardiac remodeling (i.e., left atrium and/or left ventricle enlargement), thus excluding a pre-capillary origin. The simultaneous presence of hypoxemia caused by pneumonia (type 3 PH) and chronic obstruction of the left PA caused by PA coarctation aggravated by severe thrombosis as well as mid-intimal hypertrophy of medium-caliber pulmonary arteries (type 4 PH) were likely both responsible of PH and associated severe right-sided cardiac remodeling [5,6,23].

Regarding the clinical presentation, exercise intolerance, tachypnea, and dyspnea were recurrent clinical signs in the cat described here. Respiratory distress due to generalized pulmonary hypoperfusion is often found in people with PA stenosis and it has been described in cats with severe PA stenosis [3,23,24]. In the present case, pulmonary hypoperfusion of the left lung and pneumonia were likely responsible for this clinical presentation. Pneumonia is often reported in infants affected by congenital heart disease [25] but it is rarely described in cats with similar conditions [4,26]. Severe congenital heart diseases can have serious consequences on the cardiovascular and respiratory systems, impairing the normal mechanisms of pressure and volume control. Thus, the affected patients are often hospitalized because of non-cardiac complications including infections such as pneumonia. These conditions can be an important cause of morbidity and mortality [27,28]. In the cat described here, macrophagic pneumonia associated with eosinophilic granular material and lymphocytic interstitial pneumonia (LIP) were found at necropsy in the right and left lung, respectively. From a radiographic point of view, a pulmonary interstitial pattern was only visible on the right caudal lung lobe. Because two days had passed from our last examination and the cat’s death it is likely that some pulmonary lesions developed in the time frame between the radiographic examination to death, particularly those of the left lung. Among these lesions, the eosinophilic material found in the right lung could be the consequence of inhaled foreign material during the agonal period. Regarding the left lung, interstitial lung disease (ILD), including LIP, are complex pulmonary disorders with multifaceted etiology and whose classification has been repeatedly revised in human medicine. A classification scheme of ILD in dogs and cats has been recently proposed recognizing three major groups: idiopathic interstitial pneumonia, ILD secondary to known causes, and miscellaneous ILDs [29]. According to this proposed classification, LIP is included among idiopathic interstitial pneumonias. Lymphocytic interstitial pneumonia is a diffuse lymphoid proliferation in the lung that has been rarely reported in cats and was described in five cats with FIV infection [30]. The development of this type of pneumonia in the cat of this report could be attributable to hypoperfusion, use of different drugs, or idiopathic causes, but the precise etiology only remains speculative.

Balloon angioplasty and stent procedures are the main treatment options for the correction of PA coarctation or distal PA stenosis in humans [31]. In cats, balloon angioplasty has been attempted in two animals with right PA coarctation and concomitant left PA interruption associated with severe respiratory signs [4,32]. In one cat, the procedure was successful in resolving the clinical signs, whereas the other animal died intraoperatively. In the cat reported here, palliative therapy was administered for the treatment of pneumonia and PH, but it failed to resolve the clinical signs.

## 4. Conclusions

In conclusion, we reported here the unique association of a rare congenital cardiovascular disease (i.e., left PA coarctation), pneumonia, pre-capillary PH, and severe right-sided cardiac remodeling in a cat. Although a complete diagnostic work-up was not performed, the presumptive diagnosis derived from radiographic and echocardiographic examination was confirmed at necropsy. 

## Data Availability

The data presented in this study are available on request from the corresponding author.
